# Norcantharidin ameliorates the development of murine lupus via inhibiting the generation of IL-17 producing cells

**DOI:** 10.1038/s41401-021-00773-7

**Published:** 2021-09-22

**Authors:** Li-jun Du, Yu-xiang Feng, Zhi-xing He, Lin Huang, Qiao Wang, Cheng-ping Wen, Yun Zhang

**Affiliations:** Institute of Basic Research in Clinical Medicine, College of Basic Medical Science, Zhejiang Chinese Medical University, Hangzhou, 310053 China

**Keywords:** systemic lupus erythematosus, norcantharidin, DN T cell proliferation, Th17 cell differentiation, IL-17, STAT3, MRL/*lpr* mice

## Abstract

Systemic lupus erythematosus (SLE) is a devastating autoimmune disorder associated with severe organ damage. The abnormality of T cell apoptosis is considered as an important pathogenetic mechanism of SLE. Norcantharidin (NCTD), a derivative of Cantharidin, is an efficacious anti-cancer drug by inhibiting cell proliferation and inducing cell apoptosis. Besides, NCTD has also been proved to protect the function of kidneys, while damaged renal function is the most important predictor of morbidity and mortality in SLE. All these suggest the potential effects of NCTD in SLE treatment. In this study we investigated whether NCTD exerted therapeutic effects in a mouse SLE model. Lupus prone female MRL/*lpr* mice were treated with NCTD (1, 2 mg·kg^−1^·d^−1^, ip) for 8 weeks. We showed that NCTD administration significantly decreased mortality rate, diminished the expression of anti-dsDNA IgG antibody, a diagnostic marker for SLE, as well as restored renal structure and function in MRL/*lpr* mice. Moreover, NCTD administration dose-dependently inhibited lymphoproliferation and T cell accumulation in the spleens of MRL/*lpr* mice. We further revealed that NCTD specifically inhibited DN T cell proliferation and Th17 cell differentiation both via blocking activation of signal transducer and activator of transcription 3 (STAT3) signaling pathway. On the other hand, NCTD did not affect T cell apoptosis in MRL/*lpr* mice. Taken together, our data suggest that NCTD may be as a promising therapeutic drug through targeting T cells for the treatment of SLE.

## Introduction

Systemic lupus erythematosus (SLE) is a complicated autoimmune disease, manifested by autoantibody accumulation, systemic inflammation and immune complex deposits in multi-organs, especially the kidneys [1]. Recent studies support that T cells serve as the commander in SLE pathogenesis with orchestrating not only B cell activation for autoantibody production but also the modulation and differentiation of T helper (Th) cells and inflammation cytokine infiltration in target organs such as kidneys, finally resulting in systemic damage [2, 3]. Considerable evidences show that abnormal accumulation and activation of T cells have been closely related to the immunopathogenesis of SLE [4]. As a result, the development of new therapeutic agents for SLE targeting T cell hyperplasia is promising and necessary.

Interleukin-17 (IL-17/IL-17A) has been reported to play a central pathogenic role in the development of SLE [5, 6]. Patients with SLE show higher levels of IL-17 in serum accompanied with increased number of IL-17 producing T cells [7–9]. During SLE, IL-17 may mediate local tissue damage by inducing other inflammatory chemokines and cytokines to promote the recruitment of immune cells such as monocytes and neutrophils [10, 11]. In addition, IL-17 has been demonstrated to have a synergy with B-cell activating factor (BAFF) to promote B cell proliferation and autoantibody production [12]. During SLE progress, double-negative (CD3^+^ CD4^−^CD8^−^, DN) T cells as well as Th17 cells are the main source of IL-17 [9, 13]. DN T cells invade into multi-organs of SLE patients and contribute to loss of tolerance. In addition, DN T cells promote B cell differentiation, induce the production of autoantibodies as well as the secretion of pro-inflammatory cytokines including IFN-γ and IL-17 [14, 15]. Th17 cells, a T cell subset derived from CD4^+^ T cells, also secret IL-17, IL-21, and IL-22 to regulate the inflammatory process of SLE. It has been proved that the number of IL-17 producing cells and serum IL-17 concentration are positively related to SLE activity in SLE patients [16], making them attractive therapeutic targets for SLE [17].

Signal transducer and activator of transcription 3 (STAT3), a pivotal regulator of T cell responses [18], has been confirmed to positively regulate IL-17 expression and Th17 differentiation [19–21]. Besides, previous studies have indicated that loss of STAT3 in T cells abrogated lupus nephritis (LN) [22] and STAT3 pathway was involved in DN T cell proliferation [23]. As a result, STAT3 signaling represents a promising novel treatment of SLE for its role on IL-17 producing cells. Norcantharidin (NCTD), a low-toxic demethylated form of Cantharidin, is an anti-cancer drug routinely used in China via inhibiting proliferation and inducing apoptosis of multiple types of cancer cells [24]. Previous studies have proved that NCTD showed therapeutic effect in CIA-induced rheumatoid arthritis (RA) model by inhibiting IL-17 production [25]. Moreover, NCTD has also been demonstrated to protect renal function in different nephropathy models [26–28]. However, the role of NCTD in SLE remains unclear. In the current study, we detected the effect of NCTD as a potential therapeutic agent for SLE treatment via targeting STAT3 pathway.

MRL/*lpr* mice, with the mutation in the *Fas* gene, exhibit the expansion of IL-17 producing cells and spontaneously develop syndromes resembling human SLE [29]. As a result, MRL/*lpr* mice are good surrogates for studying this disease. In our study, we treated female MRL/*lpr* mice with NCTD or vehicle from 12 weeks to 20 weeks and found that NCTD-treated MRL/*lpr* mice showed significantly alleviative lupus-like syndrome including improved survival rate, decreased production of autoantibodies, improved kidney function and diminished LN. Further research suggested that NCTD impaired DN T cell proliferation and Th17 cell differentiation via blocking the activation of STAT3, while NCTD has no effects on T cell apoptosis. Our research reveals NCTD may be as a promising therapeutic drug for SLE treatment.

## Materials and methods

### Mice

MRL/*lpr* and MRL/MpJ female mice (4–5 weeks) were purchased from SLRC Experimental Animals Co. Ltd. (Shanghai, China). From 12 weeks, NCTD (Sigma-Aldrich, St. Louis, MO, USA) was dissolved and administered by intraperitoneal injection into MRL/*lpr* or MRL/MpJ mice at 1 mg/kg or 2 mg/kg every day for 8 weeks. The dosage of NCTD was established according to previous studies [30–32]. The death of these mice was recorded every day. We collected 100 μL of blood without anticoagulants per mouse once a week from the submandibular vein. Then the serum was obtained and diluted to detect the concentrations of anti-dsDNA. At 20 weeks, the mice were narcotized for collecting blood without anticoagulants (1 mL/mouse) by heart punctures to measure the level of anti-dsDNA and cytokines. Then the mice were sacrificed and the spleens as well as kidneys were collected. The weight of spleens was measured as well as total splenocyte number was assessed by the Cedex XS cell analysis system (Roche, Mannheim, Germany). All animal experiments were carried out according to the guidelines of the National Institutes of Health (NIH) Guide for the Care and Use of Laboratory Animals. The experimental procedures were reviewed and approved by the Institutional Animal Care and Use Committee of Zhejiang Chinese Medical University.

### Renal histology and immunofluorescence (IF)

Kidneys were obtained from exsanguinated mice, immediately fixed with 4% formalin and then embedded in paraffin according to standard procedures. Sections (5 μm) were mounted on slides for hematoxylin and eosin (H&E) and periodic acid-Schiff (PAS) staining to evaluate the morphology changes and inflammation level in kidneys. Renal pathology was scored by an experienced pathologist blinded to the treatments according to previously described methods [33]. Besides, immune complex (IgG) deposition was assessed with IgG-FITC antibody (Abcam Ltd., Cambridge, UK) in accordance with a previous protocol [34] and the fluorescence intensity was quantified by ImageJ.

### Urinalysis

Urine was manually harvested from 19 weeks. Fresh urine samples were centrifuged at 1500 r/min for 10 min at 4 °C and pooled for each mouse followed by storing at −80 °C until use. Levels of total protein, albumin and creatine in urine were detected with commercially available kits according to the manufacturer’s instructions (Dia Sys Diagnostic Systems GmbH, Holzheim, Germany).

### Bio-Plex cytokine assay

Serum samples were collected from whole blood without anticoagulants at 3000 r/min for 10 min at 4 °C. Cytokines in serum were quantified with the Bio-Plex Pro^TM^ Mouse Cytokine 23-plex Assay (Bio-Rad Laboratories,Inc., Hercules, CA, USA) according to the manufacturer’s instructions. These measured cytokines include IL-1α, IL-1β, IL-2, IL-3, IL-4, IL-5, IL-6, IL-9, IL-10, IL-12 (p40), IL-12 (p70), IL-13, IL-17, IFN-γ, TNF-α, MCP-1, Eotaxin, G-CSF, GM-CSF, KC, MIP-1α, MIP-1β, and RANTES. A parallel standard curve was constructed for each cytokine.

### Enzyme-linked immunosorbent assay (ELISA)

The concentrations of anti-dsDNA (SHIBAYAGI Co. Ltd, Shibukawa, Japan), IFN-γ, IL-17, IL-22, IL-23, IL-4, and IL-10 (Thermo Fisher Scientific, Waltham, MA, USA) in serum were determined with corresponding ELISA kits according to the manufacturer’s instructions. In addition, the culture supernatant of Th17 cells and DN T cells was collected, then IL-17 production was detected as described above.

### Flow cytometry

To measure T/B cell percentage, single-cell suspensions of spleens were prepared and stained with PE anti-mouse CD3 and FITC anti-mouse CD19 antibodies (Thermo Fisher Scientific) followed by FACS analysis using an FC 500 MC system (Beckman Coulter, Fullerton, CA, USA). To analyze the T cell subsets in spleens, single-cell suspensions were re-stimulated with 50 ng/mL PMA (Sigma-Aldrich), 1 μg/mL ionomycin (Sigma-Aldrich) and 10 μg/mL Brefeldin A (Sigma-Aldrich) for 5 h. Surface markers were stained with the indicated antibodies: FITC anti-mouse CD3, PE anti-mouse CD4, APC anti-mouse CD8, APC anti-mouse CD25 and APC anti-mouse CD4 (Thermo Fisher Scientific). Then cells were fixed with Fixation/permeabilization Buffer (BD Biosciences, San Jose, CA, USA), permeabilized with Perm/Wash buffer (BD Biosciences) and stained with the following antibodies: PE anti-mouse IFN-γ, FITC anti-mouse IL-17, APC anti-mouse IL-4 (Thermo Fisher Scientific) according to the manufacturer’s instructions. For Foxp3 intracellular staining, cells were treated with Foxp3 buffer (BD Biosciences), followed with FITC anti-mouse Foxp3 staining. Stained cells were evaluated using Beckman CytoFlex S system (Beckman).

### Proliferation assays in vivo

EdU assays were carried out with the Click^TM^ EdU assay kit (Beyotime Biotechnology, Shanghai, China) to evaluate cell proliferation ability in vivo. Mice were i.p. injected with 10 μg EdU per gram of body weight and were sacrificed 16 h later. Single-cell suspensions of peripheral blood and Spleen were prepared and stained with PE anti-mouse CD3 and FITC anti-mouse CD19 antibodies (Thermo Fisher Scientific). Finally, the Click-iT reaction was performed following the manufacturer’s instruction and cells were analyzed with Beckman CytoFlex S system (Beckman).

### Purification of T cells and DN T cells

Total CD3^+^ T cells isolated from spleens of MRL/*lpr* mice were obtained with negative selection using a Mouse T Cell Isolation Kit (Stem Cell Technologies Inc, Vancouver, Canada) according to the manufacturer’s instructions. On the basis, DN T cells were purified from these CD3^+^ T cells by luorescent cell sorting with anti-mouse CD3, anti-mouse CD4 and anti-mouse CD8 antibodies using BD FACSAria (BD Biosciences).

### Proliferation assay in vitro

Purified DN T cells were re-suspended in RPMI-1640 with 10% heat-inactivated FBS, 50 U/mL of penicillin and 50 U/mL of streptomycin. Then 1 × 10^5^ DN T cells, in a volume of 100 μL per well, were cultured in a 96-well plate pre-coated with 1 μg/mL anti-mouse CD3 (Biolegend, San Diego, CA, USA) overnight and then were stimulated with 0.5 μg/mL anti-mouse CD28 (Biolegend) and 20 ng/mL IL-2 (Peprotech, Rocky Hill, NJ, USA). These cells were cultured in the presence of control or NCTD (5 or 10 μg/mL) for 54 h. Another DN T cells were incubated with control, NCTD (10 μg/mL), Stattic (10 μM) or NCTD (10 μg/mL) plus Stattic (10 μM) (pre-treated with Stattic for 6 h) for 54 h. Then cells were treated with 10 μM EdU for 2 h and the Click-iT reaction was performed according to the manufacturer’s instruction. Finally, cells were analyzed with Beckman CytoFlex S system (Beckman) and the cell number was also monitored.

### Apoptosis assay

Cell apoptosis was assessed using an Annexin V-FITC/PI Staining Kit (Beyotime Biotechnology). Single-cell suspensions of peripheral blood and spleen were prepared and stained with PerCP/Cyanine5.5 anti-mouse CD3 (Biolegend), BV421^TM^ anti-mouse CD19 (Biolegend), Annexin-FITC and PI. Sorting was performed and cells were analyzed using Beckman CytoFlex S system (Beckman). Purified DN T cells isolated from MRL/*lpr* mice or CD3 T cells isolated from C57B6 mice were cultured in a 96-well plate pre-coated with 1 μg/mL anti-mouse CD3 (Biolegend) overnight and then were stimulated with 0.5 μg/mL anti-mouse CD28 (Biolegend) and 20 ng/mL IL-2 (Peprotech). These cells were cultured in the presence of control or NCTD (5 or 10 μg/mL) for 30 h and then stained with Annexin-FITC and PI. Sorting was performed and cells were analyzed using Beckman CytoFlex S system (Beckman) or BD Accuri C6 (BD Biosciences).

### Purification of CD4^+^ T cells and in vitro Th17 cell differentiation

Total CD4^+^ T cells from spleens of MRL/*lpr* mice were purified with negative selection using a Mouse T Cell Isolation Kit (Stem Cell Technologies Inc) according to the manufacturer’s instructions. The purity was determined by FACS analysis (>98% CD4^+^). For T cell differentiation, the above purified naïve CD4^+^ T cell were cultured in RPMI-1640 with 10% heat-inactivated FBS and 2 mM *L*-glutamine in the presence of 1 μg/mL anti-mouse CD3 (Biolegend) and 0.5 μg/mL anti-mouse CD28 (Biolegend), followed by treatment with or without NCTD. Then, 2 ng/mL transforming growth factor-1 (TGF-β1) (Peprotech), 20 ng/mL IL-6 (Peprotech), 10 μg/mL anti-IL-4 antibody (Biolegend) and 10 μg/mL anti-IFN-γ antibody (Biolegend) were added to drive Th17 cell polarization. After 4 days, cells were collected for FACS analysis and cell culture supernatants were also collected to detect the IL-17 production as described above.

### Immunoblotting analysis

DN T cells purified from MRL/*lpr* mice administered NCTD or control, as well as DN T cells treated with or without NCTD were homogenized in RIPA buffer (Beyotime Biotechnology) supplemented with protease and phosphatase inhibitors (Beyotime Biotechnology). Cell lysates (40 μg) were separated on SDS-PAGE and immunoblotted using antibodies against the following proteins: phospho-STAT3 (Y705) (Cell Signaling Technology, Danvers, MA, USA), STAT3 (Cell Signaling Technology), phospho-AKT (Thr308) (Cell Signaling Technology), AKT (Cell Signaling Technology) and β-actin (Sigma-Aldrich). CD4^+^ T cells treated with or without NCTD (10 μg/mL) were stimulated with IL-6 or TGF-β1 (Peprotech) in time gradient and immunoblotted using antibodies against the following proteins: phospho-STAT3 (Y705) (Cell Signaling Technology), STAT3 (Cell Signaling Technology), p-Smad2 (Cell Signaling Technology), p-Smad3 (Abcam Ltd), Smad2 (Cell Signaling Technology), Smad3 (Abcam Ltd) and β-actin (Sigma-Aldrich).

### Statistical analysis

All results were expressed as the mean ± SEM and were determined by *t* test or two-way ANOVA with GraphPad Prism 8, as appropriate. If ANOVA was significant, individual differences were determined with Tukey post-test. In our studies, *P* values < 0.05 were considered statistically significant.

## Results

### NCTD treatment significantly prevents SLE development of MRL/*lpr* mice

**Fig. 1:**
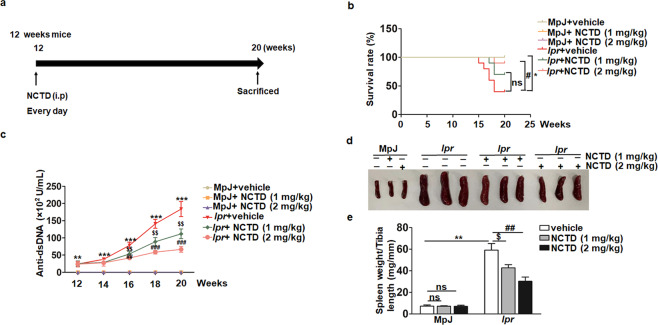
NCTD treatment significantly prevents SLE development of MRL/*lpr* mice. **a** MRL/*lpr* and MRL/MpJ female mice were randomly divided into three groups and administered vehicle control, 1 or 2 mg/kg NCTD via i.p. every day for 8 weeks as the indicated scheme. *n* = 5 (MRL/MpJ group) or 10 (MRL/*lpr* group). **b** Survival curve of 12- to 20-week-old MRL/MpJ and MRL/*lpr* mice subjected to either control or NCTD treatment. *n* = 5 (MRL/MpJ group) or 10 (MRL/*lpr* group). *P *<* 0.05, MRL/*lpr* *+* vehicle vs. MRL/MpJ + vehicle; ^#^*P* *<* 0.05, MRL/*lpr* + NCTD (2 mg/kg) vs. MRL/*lpr* + vehicle. **c** The serum was collected from 12 week and level of anti-dsDNA antibody was monitored, *n* = 3 (MRL/MpJ group) or 4 (MRL/*lpr* group). **P *<* 0.01, ***P *<* 0.001, MRL/*lpr* + vehicle vs. MRL/MpJ + vehicle; ^$$^*P* *<* 0.01, MRL/*lpr* + NCTD (1 mg/kg) vs. MRL/*lpr* + vehicle; ^##^*P* *<* 0.01, ^###^*P* *<* 0.001, MRL/*lpr* + NCTD (2 mg/kg) vs. MRL/*lpr* + vehicle. **d** Representative spleens from MRL/MpJ and MRL/*lpr* mice subjected to either vehicle or NCTD treatment. **e** Spleen weight to tibia length ratios of MRL/MpJ and MRL/*lpr* mice subjected to either vehicle or NCTD treatment (1 and 2 mg/kg). *n* = 4 (MRL/*lpr* treated with vehicle control), 5 (MRL/MpJ group), 7 (MRL/*lpr* treated with 1 mg/kg NCTD), or 9 (MRL/*lpr* treated with 2 mg/kg NCTD). **P < 0.01, MRL/*lpr* + vehicle vs. MRL/MpJ + vehicle; ^$^*P* *<* 0.05, MRL/*lpr* + NCTD (1 mg/kg) vs. MRL/*lpr* + vehicle; ^##^*P* *<* 0.01, MRL/*lpr* + NCTD (2 mg/kg) vs. MRL/*lpr* + vehicle.

To well define the role of NCTD in SLE, lupus prone female MRL/*lpr* mice were used. According to previous research, MRL/*lpr* mice spontaneously develop symptoms of SLE similar to human SLE approximately from 12 weeks [35]. As a result, MRL/MpJ and MRL/*lpr* female mice were treated with vehicle or NCTD (1 and 2 mg/kg) every day from 12 weeks of age to 20 weeks (Fig. 1a). At the stage, the death of mice was recorded. Survival curves displayed that NCTD-treated MRL/*lpr* group showed higher survival rates compared with the control group, while all MRL/MpJ groups showed no death (Fig. 1b). Besides, the level of anti-dsDNA IgG antibody, a diagnostic marker for SLE [36], was notably elevated in MRL/*lpr* control compared with MRL/MpJ group during lupus development. Meanwhile, NCTD significantly inhibited the production of serum anti-dsDNA antibody in MRL/*lpr* group beginning at 16 weeks, while there was no difference between MRL/MpJ groups after NCTD treatment (Fig. 1c). Moreover, spleens isolated from MRL/*lpr* treated with NCTD were substantially diminished (Fig. 1d), with lower spleen weights (Fig. 1e). Altogether, our data suggest that NCTD is able to alleviate lupus symptoms and improve survival rate of MRL/*lpr* mice.

### Restored renal structures and functions in NCTD-treated MRL/*lpr* mice

**Fig. 2:**
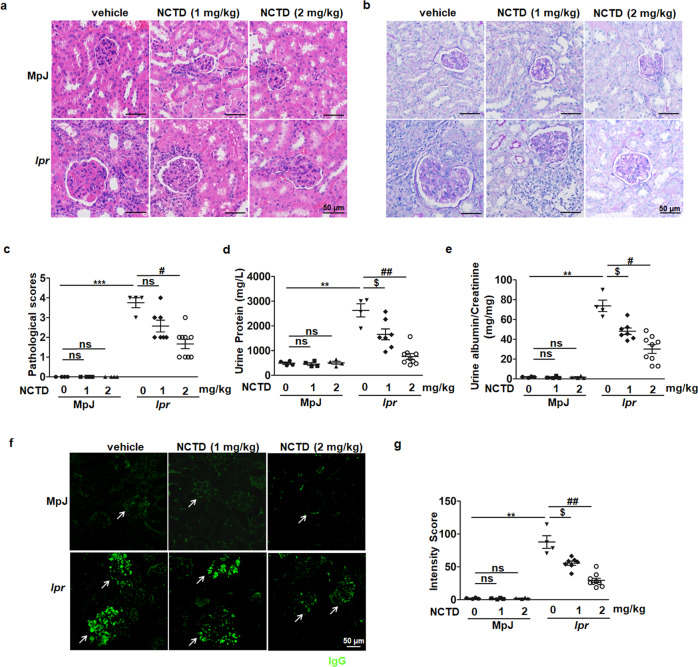
NCTD administration attenuated renal damage in MRL/*lpr* mice. **a**, **b** Representative images of H&E and PAS staining from kidneys of 20-week-old MRL/MpJ and MRL/*lpr* mice treated with vehicle control or NCTD. Scale bar = 50 µm, *n* = 4 (MRL/MpJ group and MRL/*lpr* treated with vehicle control group), 7 (MRL/*lpr* treated with 1 mg/kg NCTD), or 9 (MRL/*lpr* treated with 2 mg/kg NCTD) (The same below). **c** Histologic damage of kidneys was evaluated with pathological scores. The score of each mouse was calculated from the total scores of observed five glomeruli. *n* = 4, 7, or 9 mice/group. ***P *<* 0.001, MRL/*lpr* + vehicle vs. MRL/MpJ + vehicle; ^#^*P* *<* 0.05, MRL/*lpr* + NCTD (2 mg/kg) vs. MRL/*lpr* + vehicle. **d** Decreased urine protein level was found in NCTD-treated MRL/*lpr* mice. *n* = 4, 7, or 9 mice/group. **P < 0.01, MRL/*lpr* + vehicle vs. MRL/MpJ + vehicle; ^$^*P<* 0.05, MRL/*lpr* + NCTD (1 mg/kg) vs. MRL/*lpr* + vehicle; ^##^*P* *<* 0.01, MRL/*lpr* + NCTD (2 mg/kg) vs. MRL/*lpr* + vehicle. **e** Albumin and creatinine in urine were analyzed and the ratio of albumin to creatinine was decreased by NCTD treatment in MRL/*lpr* mice. *n* = 4, 7, or 9 mice/group. **P *<* 0.01, MRL/*lpr* + vehicle vs. MRL/MpJ + vehicle; ^$^*P* *<* 0.05, MRL/*lpr* + NCTD (1 mg/kg) vs. MRL/*lpr* + vehicle; ^#^*P* *<* 0.05, MRL/*lpr* + NCTD (2 mg/kg) vs. MRL/*lpr* + vehicle. **f** IgG deposition in the glomeruli was revealed by immunofluorescence staining of IgG (green). White arrows indicate glomeruli. Representative images were shown. Scale bar = 50 µm, *n* = 4, 7, or 9 mice/group. **g** The mean fluorescence intensity of IgG accumulation was assessed by ImageJ. *n* = 4, 7, or 9 mice/group. **P *<* 0.01, MRL/*lpr* + vehicle vs. MRL/MpJ + vehicle; ^$^*P* *<* 0.05, MRL/*lpr* + NCTD (1 mg/kg) vs. MRL/*lpr* + vehicle; ^##^*P* *<* 0.01, MRL/*lpr* + NCTD (2 mg/kg) vs. MRL/*lpr* + vehicle.

Next, we analyzed the renal structure and function of MRL/*lpr* mice. Histologically, NCTD significantly suppressed the progressive crescent glomerulonephritis and inflammatory cell infiltration of kidneys in MRL/*lpr* mice, which were assessed by histologic examination of H&E and PAS-stained kidney sections (Fig. 2a, b). According to the composite score integrating glomerular deposition, glomerular crescent formation, immune cell infiltration and endocapillary proliferation of PAS-stained sections, we found that NCTD prevented renal damage of MRL/*lpr* mice and the inhibitory effect of 2 mg·kg^−1^·d^−1^ NCTD was much better than that of 1 mg·kg^−1^·d^−1^ (Fig. 2b, c). Moreover, to monitor the effects of NCTD on renal function, urine samples were collected to measure the total protein and albumin: creatinine ratio. As expected, we observed that NCTD-treated MRL/*lpr* mice showed a notable decrease in total protein (Fig. 2d), as well as a reduction in albumin: creatinine ratio (Fig. 2e) in urine, indicating a prominent restoration in renal function for the use of NCTD. Considering the immune complex deposition as an important pathological mechanism in LN, IgG deposit in kidneys was evaluated via IgG staining. As shown in Fig. 2f, g, IgG deposit in the glomerulus was remarkably inhibited after NCTD administration in MRL/*lpr* mice. Collectively, these results clearly define that NCTD prevents renal damage in MRL/*lpr* mice.

### Decreased lymphoproliferation and T cell accumulation in MRL/*lpr* mice treated with NCTD

**Fig. 3:**
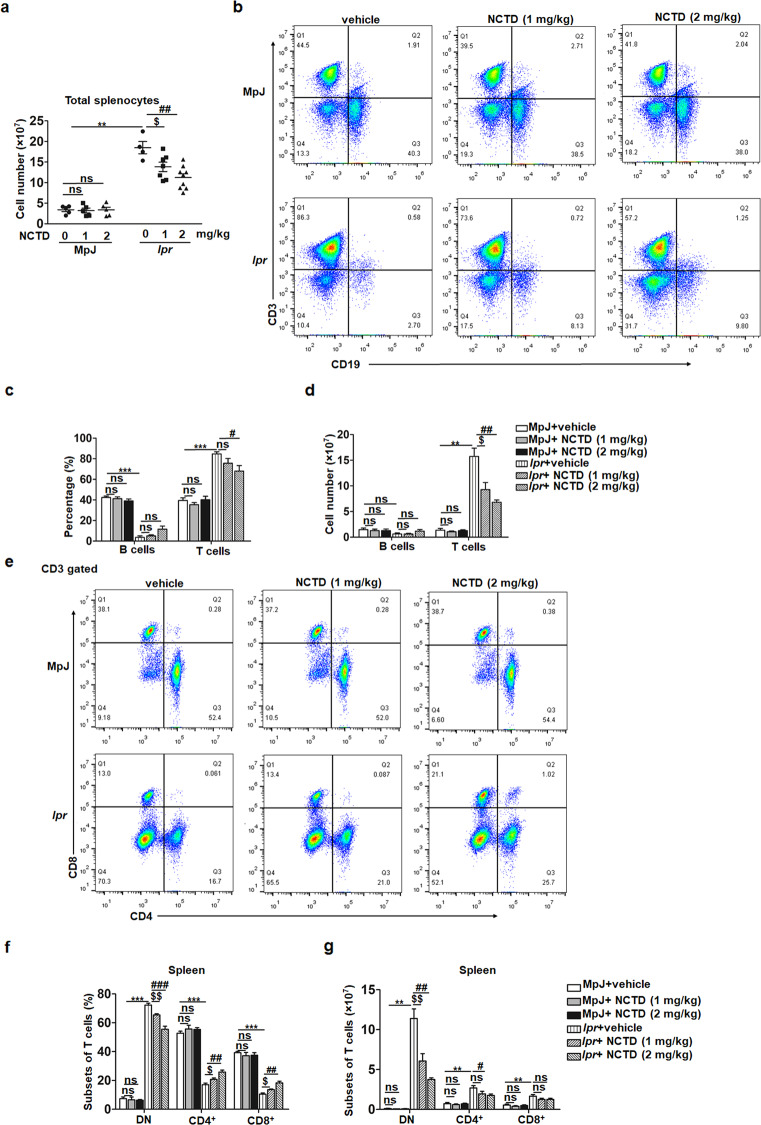
Reduced lymphoproliferation and T cell accumulation after NCTD administration. **a** Decreased splenocyte number was found in MRL/*lpr* mice treated with NCTD. *n* = 4, 7, or 9 mice/group. **P *<* 0.01, MRL/*lpr* + vehicle vs. MRL/MpJ + vehicle; ^$^*P* *<* 0.05, MRL/*lpr* + NCTD (1 mg/kg) vs. MRL/*lpr* + vehicle; ^##^*P* *<* 0.01, MRL/*lpr* + NCTD (2 mg/kg) vs. MRL/*lpr* + vehicle. **b** Percentage of T cells and B cells from spleens of MRL/*lpr* and MRL/MpJ mice was analyzed via flow cytometry. *n* = 3 (MRL/MpJ group) or 4 (MRL/*lpr* group). **c**, **d** Statistical results of the percentage and number of B cells and T cells. *n* = 3 (MRL/MpJ group) or 4 (MRL/*lpr* group). **P *<* 0.01, ***P *<* 0.001, MRL/*lpr* + vehicle vs. MRL/MpJ + vehicle; ^$^*P* *<* 0.05, MRL/*lpr* + NCTD (1 mg/kg) vs. MRL/*lpr* + vehicle; ^#^*P* *<* 0.05, ^##^*P* *<* 0.01, MRL/*lpr* + NCTD (2 mg/kg) vs. MRL/*lpr* + vehicle. **e**–**g** Flow cytometric analysis of CD3 gated cells to identify T cell subsets including DN (CD4^−^CD8^−^), CD4^+^ and CD8^+^ cells from spleens in 20-week-old MRL/MpJ and MRL/*lpr* mice treated with vehicle control or NCTD. Then the percentage and total number of T cell subsets were quantified according to the results of flow cytometry. *n* = 3 (MRL/MpJ group) or 4 (MRL/*lpr* group). **P *<* 0.01, ***P *<* 0.001, MRL/*lpr* + vehicle vs. MRL/MpJ + vehicle; ^$^*P* *<* 0.05, ^$$^*P* *<* 0.01, MRL/*lpr* + NCTD (1 mg/kg) vs. MRL/*lpr* + vehicle; ^#^*P* *<* 0.05, ^##^*P* *<* 0.01, ^###^*P* *<* 0.001, MRL/*lpr* + NCTD (2 mg/kg) vs. MRL/*lpr* + vehicle.

MRL/*lpr* mice develop massive lymphoproliferation, accompanied with splenomegaly and finally result in autoimmune response [35, 37]. A significant decrease in the total number of splenocytes in NCTD-treated mice (Fig. 3a) is consistent with the results of spleen weight (Fig. 1e), suggesting a reduction in lymphoproliferation. As a result, we explored the effect of NCTD on lymphoproliferation in spleens by analyzing the T and B cell populations using flow cytometry. Results showed that there were no significant differences in the percentage and number of B cells between NCTD and vehicle-treated MRL/*lpr* mice, while the proportion and number of T cells were reduced in NCTD groups compared to control group in MRL/*lpr* mice (Fig. 3b-d). On this basis, further analyses found that the percentage and number of DN T cells were remarkably decreased by NCTD treatment (Fig. 3e-g). Besides, despite the improvement of CD4^+^ and CD8^+^ T cell proportion, the number of CD4^+^ T cells was reduced in NCTD-treated group, while the number of CD8^+^ T cells remained unchanged in MRL/*lpr* mice (Fig. 3e-g). These results suggest that NCTD suppresses T cell hyperproliferation and reduces the generation of pathogenic T cells in MRL/*lpr* mice.

### NCTD treatment blocks STAT3-mediated T cell proliferation

**Fig. 4:**
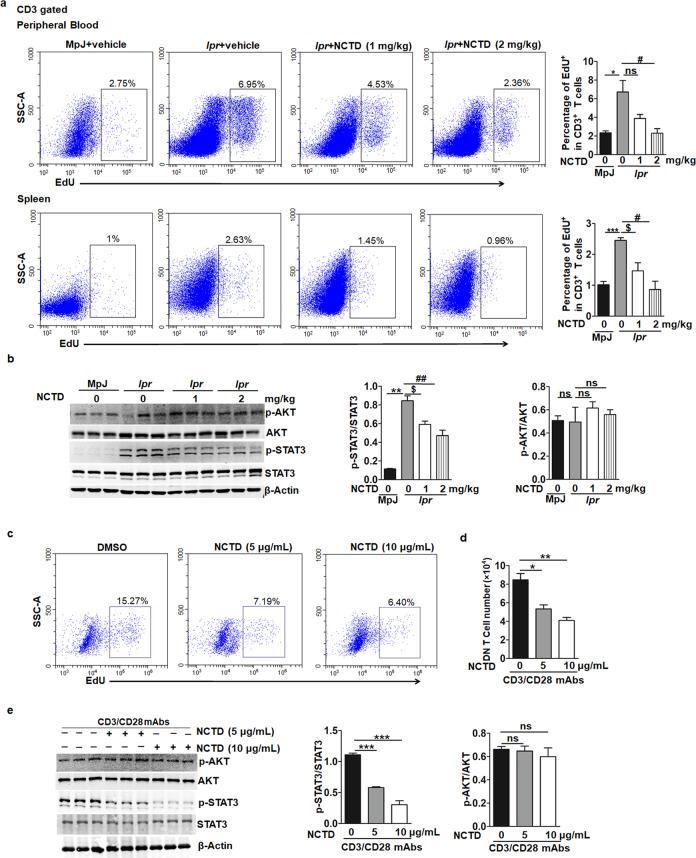
NCTD treatment blocked DN T cell proliferation by inhibiting STAT3 activation. **a** Flow cytometric analysis of CD3 gated cells to identify T cell proliferation of peripheral blood and spleen. *n* = 3 mice/group. *P *<* 0.05, ***P < 0.001, MRL/*lpr* + vehicle vs. MRL/MpJ + vehicle; ^$^*P* *<* 0.05, MRL/*lpr* + NCTD (1 mg/kg) vs. MRL/*lpr* + vehicle; ^#^*P* *<* 0.05, MRL/*lpr* + NCTD (2 mg/kg) vs. MRL/*lpr* + vehicle. **b** Western blot assay and densitometry analysis indicating the phosphorylation of STAT3 (Y705) was blocked while the activation of AKT remained unchanged in DN T cells isolated from the spleens of NCTD-treated MRL/*lpr* mice. β-Actin was used as loading control. Data were representative of three independent experiments. **P < 0.01, MRL/*lpr* + vehicle vs. MRL/MpJ + vehicle, ^$^*P* *<* 0.05, MRL/*lpr* + NCTD (1 mg/kg) vs. MRL/*lpr* + vehicle; ^##^*P* *<* 0.01, MRL/*lpr* + NCTD (2 mg/kg) vs. MRL/*lpr* + vehicle. **c**, **d** DN T cells purified from spleens of MRL/*lpr* mice were cultured (1 × 10^5^ cells/well) in 96-well plates for 54 h in the presence of vehicle control or NCTD (5 or 10 μg/mL) as well as incubated with anti-CD3, anti-CD28 antibodies and IL-2. Then DN T cell proliferation in vitro was detected via flow cytometry and cell number was counted. *n* = 3 /group. *P *<* 0.05, NCTD (5 μg/mL) vs. DMSO; **P *<* 0.01, NCTD (10 μg/mL) vs. DMSO. **e** DN T cells purified from spleens of MRL/*lpr* mice were cultured (1 × 10^5^ cells/well) in 96-well plates for 54 h in the presence of control or NCTD (5 or 10 μg/mL) as well as incubated with anti-CD3, anti-CD28 antibodies and IL-2. Cells were then obtained and immunoblotted with anti-p-AKT, anti-AKT, anti-p-STAT3, and anti-STAT3 antibodies. Results showed that NCTD suppressed STAT3 activation and had no influence on AKT activation in DN T cells. β-Actin was used as loading control. Data were representative of three independent experiments. ***P *<* 0.001, NCTD (5 or 10 μg/mL) vs. DMSO.

According to our previous data, the generation of T cells especially DN T cells was inhibited after NCTD treatment. NCTD has been well-identified to inhibit cell proliferation and promote apoptosis [38, 39]. As a consequence, we firstly monitored the proliferation and apoptosis of T cells. Results indicated that 1 mg/kg NCTD slightly inhibited T cell proliferation, while 2 mg/kg NCTD significantly impaired T cell proliferation in peripheral blood. Meanwhile, NCTD (1 and 2 mg/kg) notably blocked T cell proliferation in spleens (Fig. 4a). At the same time, T cell apoptosis including early and late apoptosis has been detected and no significant difference was found in MRL/*lpr* groups (Supplementary Fig. S1a). Furthermore, we also found that B cell proliferation and apoptosis including early and late apoptosis were normal in NCTD-treated MRL/*lpr* mice (Supplementary Fig. S2a, b), which is consistent with previous results (Fig. 3b-d). PI3K/AKT signaling is the main target pathway that NCTD regulates cell proliferation and apoptosis [38, 40]. As a result, we firstly detected the AKT activation in DN T cells and results showed that AKT activation was not affected after NCTD administration (Fig. 4b). STAT3 signaling has been demonstrated to directly regulate T cell survival and proliferation [41], so we also measured the phosphorylation of STAT3 and found NCTD notably blocked STAT3 phosphorylation (Fig. 4b). To further confirm the role of NCTD in mediating T cell proliferation, we purified DN T cells from spleens of MRL/*lpr* mice and treated with or without NCTD. We found that the proliferation of DN T cell was inhibited after NCTD treatment (Fig. 4c, d). Consistently, the phosphorylation of STAT3 was also reduced in NCTD-treated DN T cells while AKT activation remained unchanged (Fig. 4e). Moreover, a specific inhibitor of STAT3 activation (Stattic) was used to treat DN T cells and results showed that the inhibition of STAT3 activation blocked the role of NCTD on DN T cell proliferation (Supplementary Fig. S3a–c), which further proved that NCTD blocked DN T cell proliferation mainly by inhibiting STAT3 activation. Taken together, these data identify that NCTD treatment impairs DN T cell proliferation by inhibiting STAT3 activation and contributes to the attenuated SLE development.

### NCTD limits inflammatory responses of MRL/*lpr* mice

**Fig. 5:**
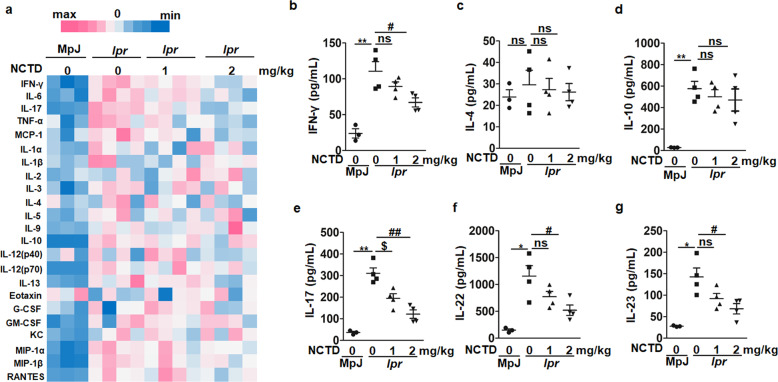
Decreased inflammation level in NCTD-treated MRL/*lpr* mice. **a** Heatmap of the inflammation cytokine level in MRL/MpJ and MRL/*lpr* mice evaluated by Bio-Plex Pro^TM^ Mouse Cytokine 23-plex Assay. *n* = 3 (MRL/MpJ group) or 4 (MRL/*lpr* group). **b**–**g** The levels of cytokines (IFN-γ, IL-4, IL-10, IL-17, IL-22, and IL-23) in serum were determined with ELISA. *n* = 3 (MRL/MpJ group) or 4 (MRL/*lpr* group). *P *<* 0.05, **P *<* 0.01, MRL/*lpr* + vehicle vs. MRL/MpJ + vehicle; ^$^*P* *<* 0.05, MRL/*lpr* + NCTD (1 mg/kg) vs. MRL/*lpr* + vehicle; ^#^*P* *<* 0.05, ^##^*P* *<* 0.01, MRL/*lpr* + NCTD (2 mg/kg) vs. MRL/*lpr* + vehicle.

During lupus, DN T cells, as a pathogenic T cell subset, contribute to the production of the key inflammatory cytokines such as IFN-γ and IL-17 [42]. We have verified that NCTD is able to reduce DN T cell generation (Figs. 3 and 4). Therefore, we hypothesized that the associated inflammatory cytokines would be reduced by NCTD treatment. As a result, we measured the levels of the common cytokines in serum of MRL/*lpr* mice at 20 weeks with the Bio-Plex Pro^TM^ Mouse Cytokine 23-plex Assay. Results indeed displayed a significant reduction of inflammatory factors in NCTD-treated MRL/*lpr* mice (Fig. 5a) including IFN-γ, IL-6, IL-17, TNF-α, and MCP-1. Further ELISA results showed that IFN-γ, IL-17, IL-22, and IL-23 were dramatically inhibited by NCTD administration, while no significant differences were observed in IL-4 and IL-10 levels between NCTD-treated and vehicle control groups (Fig. 5b–g). During SLE pathogenesis, IFN-γ is secreted by Th1 and DN T cells; IL-17 is mainly produced by Th17 and DN T cells; meanwhile, IL-23 is indispensable for the later stabilization of Th17 cells and is able to induce the production of IL-17, IL-22, IL-6, and TNF-α in Th17 cells [43–45]. Besides, IL-4 and IL-10 were respectively secreted by Th2 and Treg cells. Our data prove that NCTD treatment inhibits the inflammatory response, particularly IL-17-related cytokine-dominant inflammation in MRL/*lpr* mice.

### NCTD impaired Th17 polarization in MRL/*lpr* mice

**Fig. 6:**
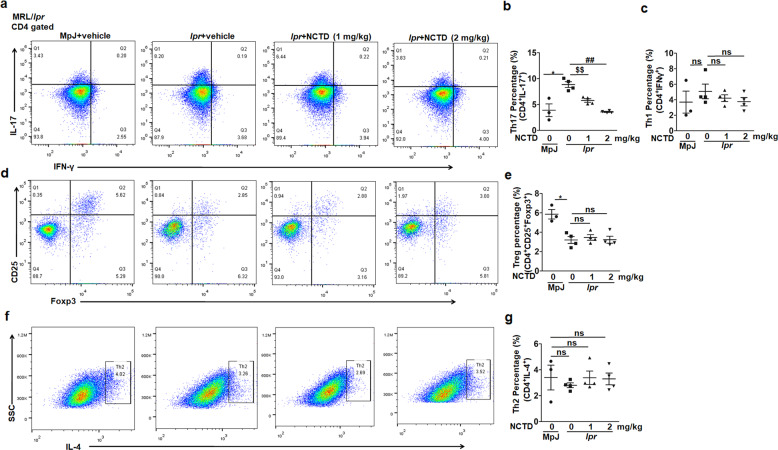
Impaired Th17 cell differentiation in vivo after NCTD treatment. Cells isolated from spleens were gated on CD4 and stained with anti-IL-17 and anti-IFN-γ antibodies. Representative flow cytometry plots (**a**) and statistical data (**b**, **c**) showing a significant decline of Th17 cell proportion and unchanged Th1 cells by NCTD treatment. *n* = 3 (MRL/MpJ group) or 4 (MRL/*lpr* group). *P *<* 0.05, MRL/*lpr* + vehicle vs. MRL/MpJ + vehicle; ^$$^*P* *<* 0.01, MRL/*lpr* + NCTD (1 mg/kg) vs. MRL/*lpr* *+* vehicle; ^##^*P* *<* 0.01, MRL/*lpr* + NCTD (2 mg/kg) vs. MRL/*lpr* + vehicle. Representative flow cytometry plots (**d**) and statistical data (**e**) showing no significant difference of Treg cell (CD4^+^ CD25^+^ Foxp3^+^) proportion by NCTD treatment. *n* = 3 (MRL/MpJ group) or 4 (MRL/*lpr* group). *P *<* 0.05, MRL/*lpr* + vehicle vs. MRL/MpJ + vehicle. Representative flow cytometry plots (**f**) and statistical data (**g**) showing no significant difference of Th2 cell (CD4^+^ IL-4^+^) proportion after NCTD treatment. *n* = 3 (MRL/MpJ group) or 4 (MRL/*lpr* group).

Our analysis of cytokines in serum has revealed that inflammatory responses was diminished after NCTD treatment (Fig. 5). Despite DN T cells, T helper cells (Th cells) are also an important source of these inflammatory cytokines. As a result, we further detected the role of NCTD on Th cell differentiation. We monitored the proportion of T cell subsets by flow cytometry and found that the percentage of Th17 cells (CD4^+ ^IL-17^+^) dramatically decreased in the NCTD-treated MRL/*lpr* mice compared to the vehicle control groups (Fig. 6a, b). However, no obvious alteration of Th1, Th2 or Treg cells was observed for the use of NCTD (Fig. 6a, c-g). Our findings indicate that NCTD administration disturbs Th17 polarization in vivo.

### NCTD suppresses Th17 cell differentiation via blocking IL-6-STAT3 pathway

**Fig. 7:**
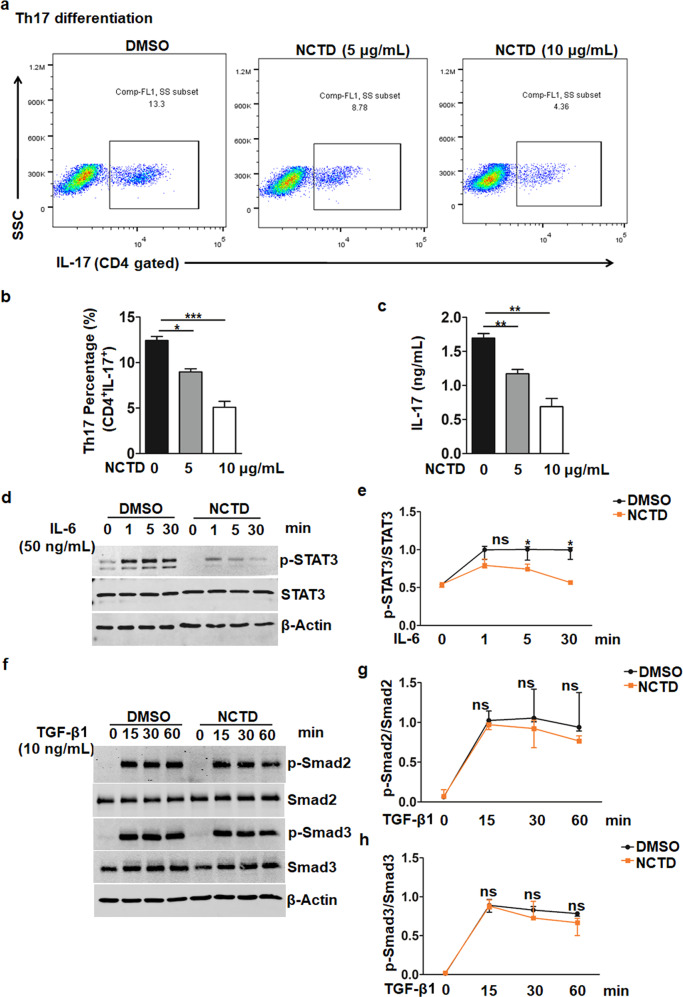
NCTD inhibited IL-6-STAT3-dependent Th17 differentiation in vitro. **a**, **b** Naïve CD4^+^ T cells from MRL/*lpr* mice were collected and treated with or without NCTD, then polarized into Th17 cells for 4 days. Representative flow cytometry plots (**a**) and statistical data (**b**) displayed an observably decreased Th17 cell (CD4^+^ IL-17^+^) percentage by NCTD treatment. *P *<* 0.05, NCTD (5 μg/mL) vs. DMSO; ***P *<* 0.001, NCTD (10 μg/mL) vs. DMSO. **c** The culture supernatants were collected and IL-17 level was monitored via ELISA. **P *<* 0.01, NCTD (5 or 10 μg/mL) vs. DMSO. **d**, **e** Western blot assay and densitometry analysis indicating NCTD blocked the phosphorylation of STAT3 (Y705) upon IL-6 stimulation in T cells. β-Actin was used as loading control. Data were representative of three independent experiments. *P *<* 0.05, NCTD vs. DMSO. **f**–**h** Western blot assay and densitometry analysis indicated NCTD had no effect on the phosphorylation of Smad2/3 upon TGF-β1 stimulation in T cells. β-Actin was used as loading control. Data were representative of three independent experiments.

To further clarify the direct role of NCTD in Th17 cell polarization, we subsequently analyzed the percentage of Th17 cells under the Th17 cell differentiation condition with or without NCTD treatment. Results showed that NCTD notably inhibited Th17 cell differentiation from CD4^+^ naïve T cells (Fig. 7a, b). We subsequently analyzed the production of IL-17 and found that the IL-17 secretion was also decreased (Fig. 7c).

Th17 cell differentiation is dependent on the activation of STAT3 induced by IL-6, which promotes the expression of retinoic acid-related orphan receptor γt (RORγt), the key transcription factor of Th17 cells [46]. Besides, in conjunction with IL-6, TGF-β1 also promotes CD4^+^ T cell differentiation into Th17 cells [47, 48]. We therefore detected the activation of IL-6-STAT3 and TGF-β1-Smad2/3 pathways. As shown in Fig. 7d, e, STAT3 phosphorylation was dramatically suppressed by NCTD administration in response to IL-6 stimulation time-dependently, while the expression of STAT3 remains unchanged. Besides, in vivo the level of IL-6 was decreased in NCTD-treated mice (Fig. 5a), which also contributed to the impaired activation of STAT3 signaling. At the same time, we analyzed the phosphorylation of Smad2 and Samd3 under TGF-β1 stimulation. Results showed that NCTD treatment had no influence on the phosphorylation and expression of Smad2/3 (Fig. 7f–h). These observations clearly define that NCTD effectively disrupts IL-6-STAT3 pathway rather than TGF-β1-Smad2/3 pathway to mediate Th17 cell polarization.

**Fig. 8:**
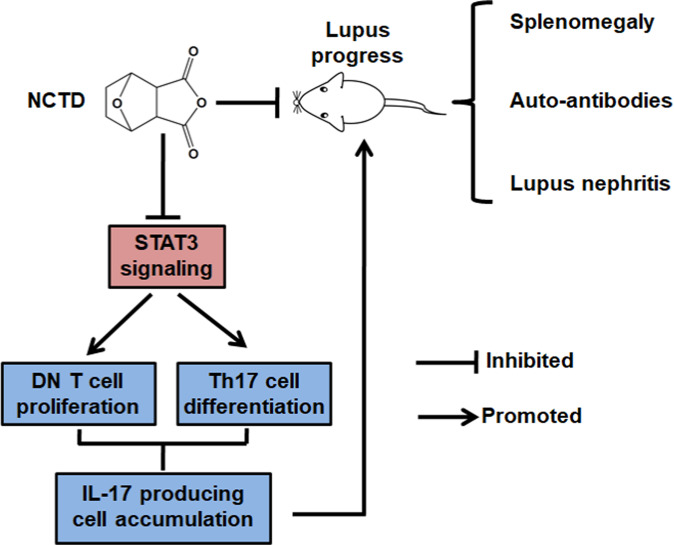
A summarization about the protective effect and underlying mechanisms of NCTD in SLE development. NCTD treatment significantly impaired DN T cell proliferation and Th17 cell differentiation via inhibiting STAT3 pathway, finally resulting in alleviated lupus symptoms in lupus mice.

As illustrated in Fig. 8, our findings clearly indicated that NCTD significantly alleviates the lupus symptoms of MRL/*lpr* mice by blocking IL-17-producing cell accumulation including DN T cells and Th17 cells. Furthermore, we found NCTD impaired DN T cell proliferation and inhibited Th17 cell differentiation both via disrupting STAT3 activation. This study suggests that NCTD may have potential clinical values in treating SLE or other IL-17-related diseases.

## Discussion

NCTD, derived from mylabris, has been used against various human cancers via inhibiting cell proliferation, inducing apoptosis or anti-angiogenesis [49–51]. Previous study has proved that NCTD is able to protect renal functions via blocking renal inflammation and proteinuria [52], while the role of NCTD in SLE remains unknown. T cells with aberrant apoptosis or hyperplasia underlie the pathology of SLE [4, 53]. Considering the function of NCTD in cell proliferation and apoptosis, we speculated that NCTD has the potential to treat SLE. Our results firstly unveiled that NCTD remarkably ameliorated SLE symptom more than LN via blocking STAT3-dependent DN T cell accumulation and Th17 cell differentiation.

We found that NCTD suppressed the progress of SLE, as illustrated by improved survival rate, suppressed autoantibody production (anti-dsDNA), alleviated systemic inflammatory response and relieved LN as well as restored renal structure and functions. Then, analysis on spleen cells showed that NCTD significantly attenuated splenomegaly accompanied with the reduced percentage and number of T cells especially DN T cells and CD4 ^+ ^T cells. Further studies proved NCTD notably inhibited T cell proliferation in vivo and in vitro. However, NCTD has no influences on T cell apoptosis as well as apoptosis-related pathways unexpectedly (Supplementary Fig. S1). We speculated the apoptosis defects of MRL/*lpr* mice for *Fas* mutation blocked the effect of NCTD on T cell apoptosis. To confirm our hypothesis, we treated T cells purified from control or MRL/*lpr* mice with NCTD and identified that NCTD significantly promoted normal T cell apoptosis (Supplementary Fig. S4) while has no influence on T cells isolated from MRL/*lpr* mice (Supplementary Fig. S5). Based on the above results, we speculated that NCTD may have stronger therapeutic effect in other models of SLE and it needs advanced research. Further studies identified that phosphorylation of STAT3 was prominently impaired by NCTD treatment, resulting in repressed DN T cell proliferation in vivo and in vitro. Moreover, we also found that the level of inflammation was attenuated after NCTD administration, particularly IFN-γ and IL-17-associated cytokines. To explore whether NCTD affects Th17 differentiation to mediate the level of IL-17-associated cytokines, we analyzed CD4 ^+ ^T cell subsets and the results exhibited that NCTD specifically inhibited Th17 cell polarization while had no effect on Th1, Th2 and Treg cell differentiation. Consistent with in vivo results, NCTD blocked Th17 cell polarization and IL-17 secretion in vitro via positively mediating IL-6-STAT3 signaling. Our findings indicate that NCTD, a traditional anti-cancer drug, is a promising therapeutic strategy clinically of SLE for its slight side-effects.

NCTD is the demethylated analog of cantharidin. They own similar functions while NCTD is less toxic [54]. Cantharidin has been proved to suppress encephalomyelitis (EAE) development mainly through limiting Th17 differentiation [47], while the role of NCTD in autoimmune disease remains unclear. IL-17, a pro-inflammatory cytokine, is involved in the pathogenesis of autoimmune diseases such as RA, EAE, and SLE [55, 56]. The deficiency of IL-17 protected mice from SLE associated with decreased autoantibodies and alleviated LN [57, 58]. Previous study has proved that NCTD inhibited IL-17 production and prevented collagen-induced arthritis [25], which suggested a potential role of NCTD on IL-17 expression. During SLE development, DN T cells and Th17 cells produce the main amounts of IL-17. Therefore, IL-17-producing cells would be potential targets for SLE treatment. Our results showed that NCTD significantly blocked the expression of IL-17-related cytokines including IL-17, IL-22, and IL-23 via limiting DN T cell accumulation and Th17 cell differentiation, while the percentage of Th1, Th2, and Treg cells remained unchanged. On the other hand, the level of IL-4 (Th2 cytokine) and IL-10 (Treg cytokine) had not been affected. In spite of the normal Th1 cell percentage, we also found that IFN-γ in serum was reduced after NCTD treatment because DN T cells were also an important source for IFN-γ except Th1 cells during SLE pathological course.

STAT3, an essential transcription factor in the pathogenesis of SLE [59], orchestrates multiple aspects of T cell function including regulating T cell activation, proliferation and Th17 cell differentiation [2, 41]. In our previous study, we have demonstrated that STAT3 signaling positively regulated DN T cell proliferation in MRL/*lpr* mice [23]. Here, we found that NCTD notably decreased STAT3 phosphorylation of DN T cells, resulting in disrupted DN T cell proliferation. Besides, specific inhibition of STAT3 activation blocked the role of NCTD on DN T cell proliferation and IL-17 production (Supplementary Fig. S3), which suggested that NCTD blocking DN T cell proliferation is mainly by inhibiting STAT3 activation. On the other hand, Th17 cell polarization was also inhibited because NCTD impaired IL-6-stimualted STAT3 activation rather than TGF-β1-dependent Smad2/3 pathway. These results were consistent with the conclusion that NCTD has no influence on Treg cell differentiation for TGF-β1-Smad2/3 signaling also responsible for Treg cell generation [60]. Further studies about the mechanism of NCTD regulating STAT3 activation may contribute to the development and application of NCTD in SLE treatment.

In our study, we found that NCTD is sufficient to dampen SLE-associated organ injury via targeting downstream auto-reactive T cells. Besides, we also proved that autoantibodies (IgG and anti-dsDNA) have been suppressed after treatment with NCTD. Despite decreased T cell generation resulting in interrupted B cell activation, we cannot rule out the direct effect of NCTD on B cells. A previous study has identified that STAT3 deficiency in B cells led to reduced B cell numbers and protected MRL/*lpr* mice from SLE [61]. As a result, we also explored B cell alterations in MRL/*lpr* mice after NCTD administration. However, we found the percentage and number of B cells exhibited a tendency of improvement while there was no statistical difference (Fig. 3) accompanied with unchanged apoptosis and proliferation of B cells in response to NCTD (Supplementary Fig. S2). Based on the above results, we infer that the distinction between T and B cells responded to NCTD was resulted from the disparity of the sensitivity to NCTD and the different cellular regulatory networks. Further research is necessary to focus on the effect of NCTD on B cell subsets, particularly plasma cells, which secrete abundant antibodies and then contribute to the pathogenesis of SLE [62]. A recent study has verified a novel function of IL-17 in plasma cell response [63], which may suggest the potential involvement of NCTD on plasma cells. In addition, T follicular helper (Tfh) cells are essential for the formation of germinal centers (GCs), where B cell affinity maturation and class switch occur leading to the generation of plasma cells and memory B cells. Aberrant activation of Tfh cells enhanced GC formation and autoantibody generation, substantially contributing to the pathogenesis of SLE [64, 65]. Besides, previous studies have suggested that STAT3 pathway is required for Tfh cell differentiation [66, 67]. Considering the significance of STAT3 pathway in Tfh cell development and STAT3 as a key target of NCTD, we speculate that Tfh cells are also the target cells of NCTD treatment in lupus development. Therefore, our further research will focus on the role of NCTD on Tfh cell and associated B cell differentiation, which can provide a more comprehensive explanation of the therapeutic mechanism of NCTD in SLE.

## Supplementary information

SuppFig 1

SuppFig 2

SuppFig 3

SuppFig 4

SuppFig 5
