# Supplementary figures and images for: Norcantharidin ameliorates the development of murine lupus via inhibiting the generation of IL-17 producing cells
Source: Acta Pharmacol Sin. 2021 Sep 22;43(6):1521–33. doi: 10.1038/s41401-021-00773-7 (PMC9159996; doi:10.1038/s41401-021-00773-7)

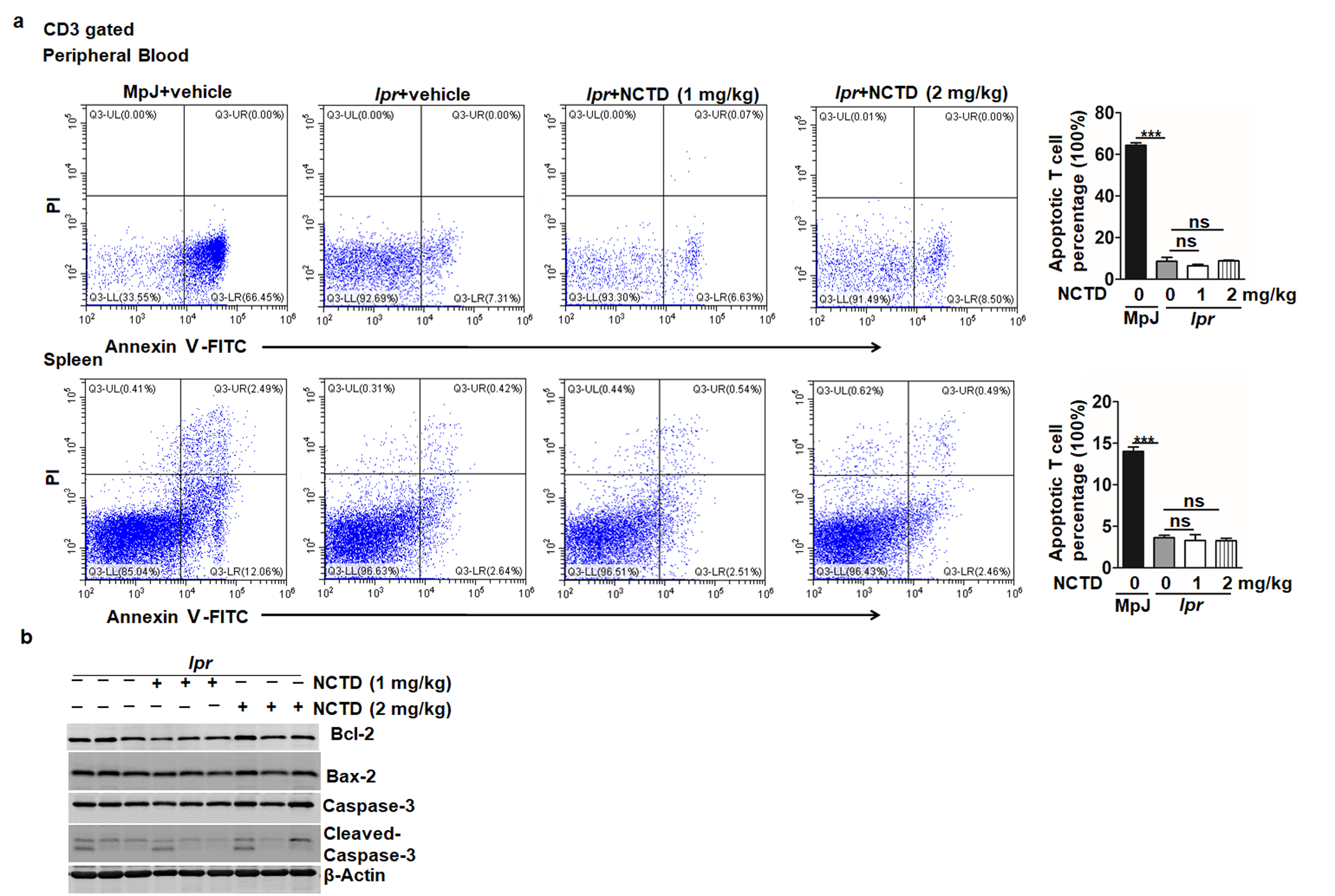

Supplement: Supplementary file 1 — SuppFig 1 [file 41401_2021_773_MOESM1_ESM.tiff]

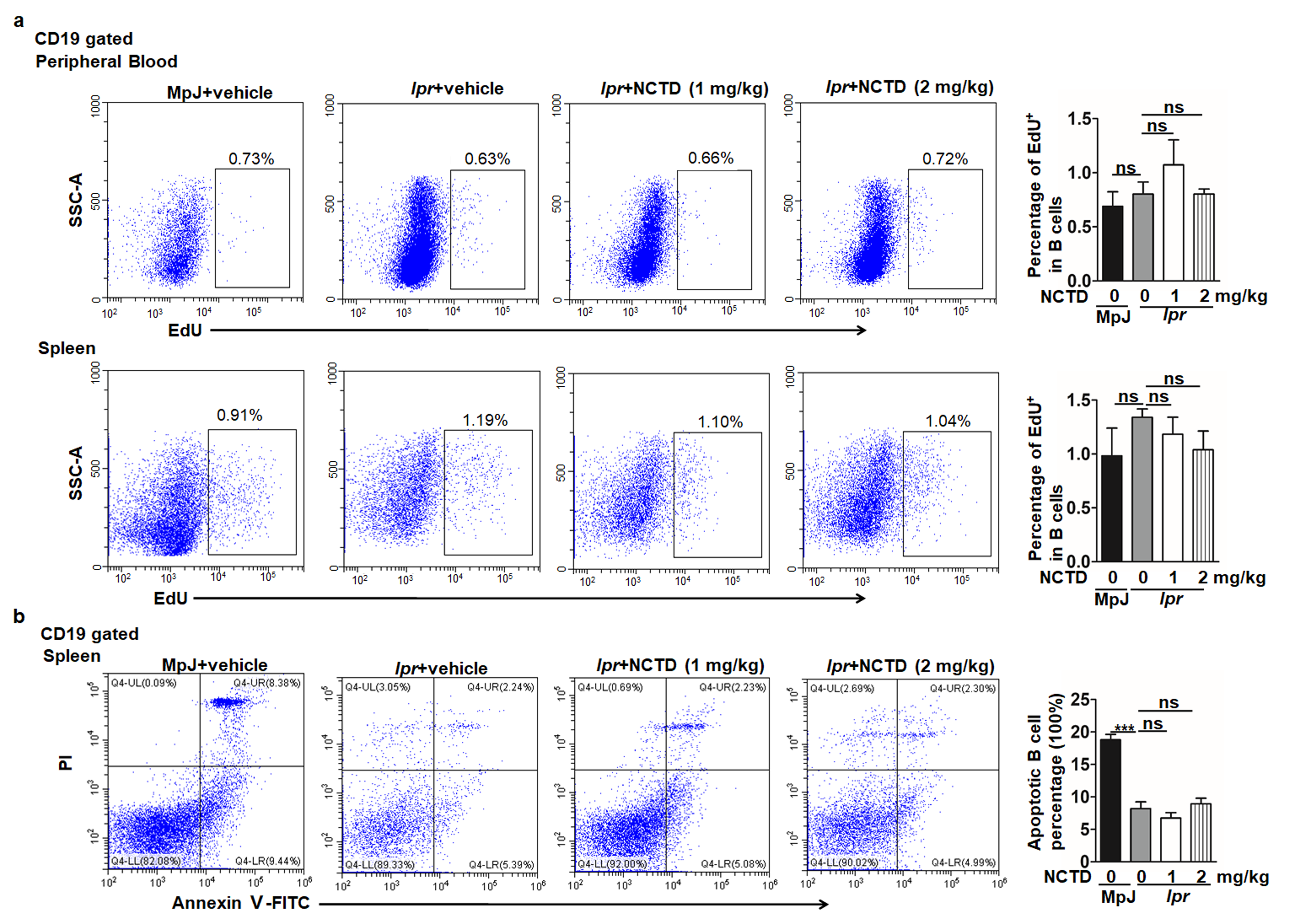

Supplement: Supplementary file 2 — SuppFig 2 [file 41401_2021_773_MOESM2_ESM.tif]

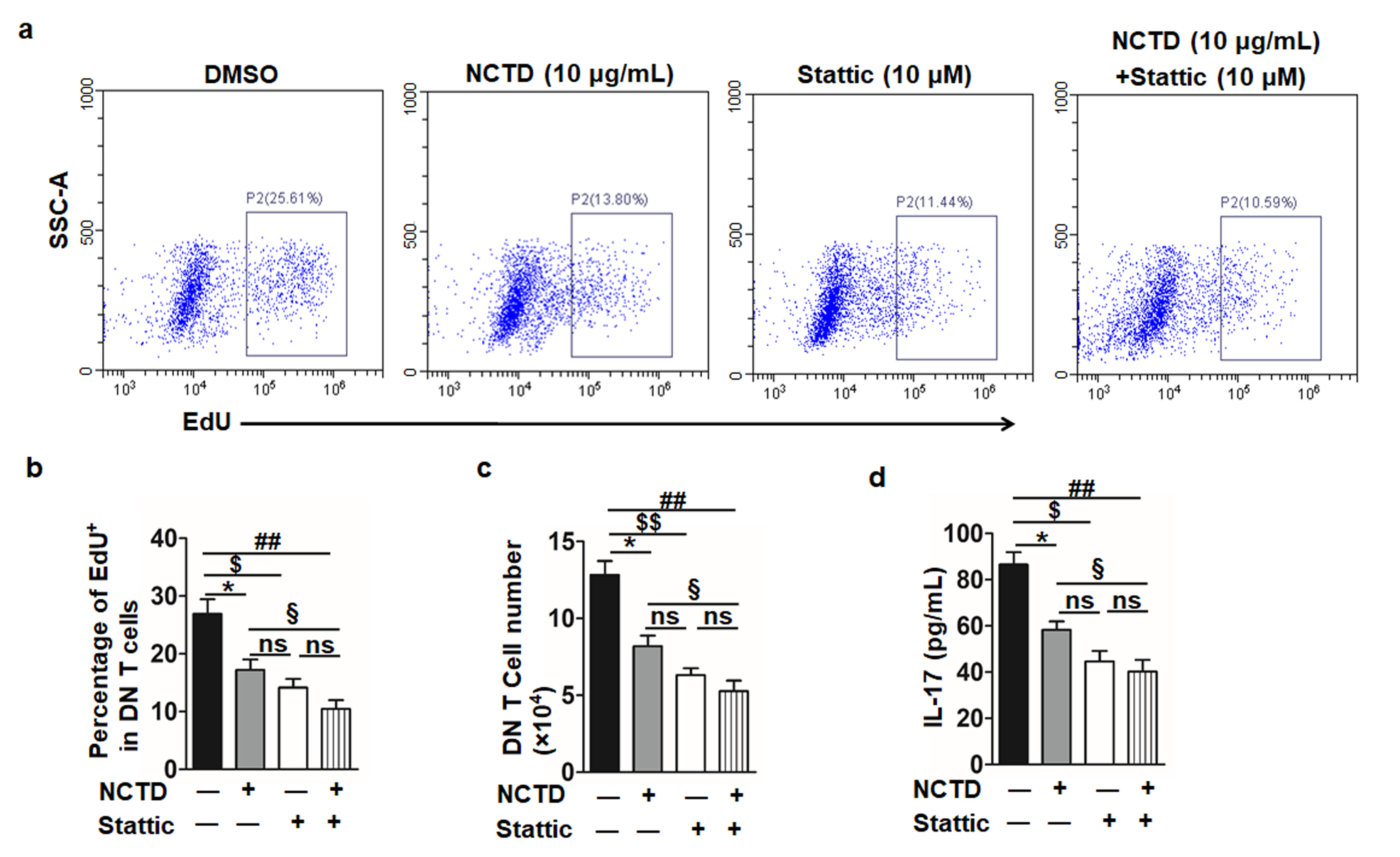

Supplement: Supplementary file 3 — SuppFig 3 [file 41401_2021_773_MOESM3_ESM.tif]

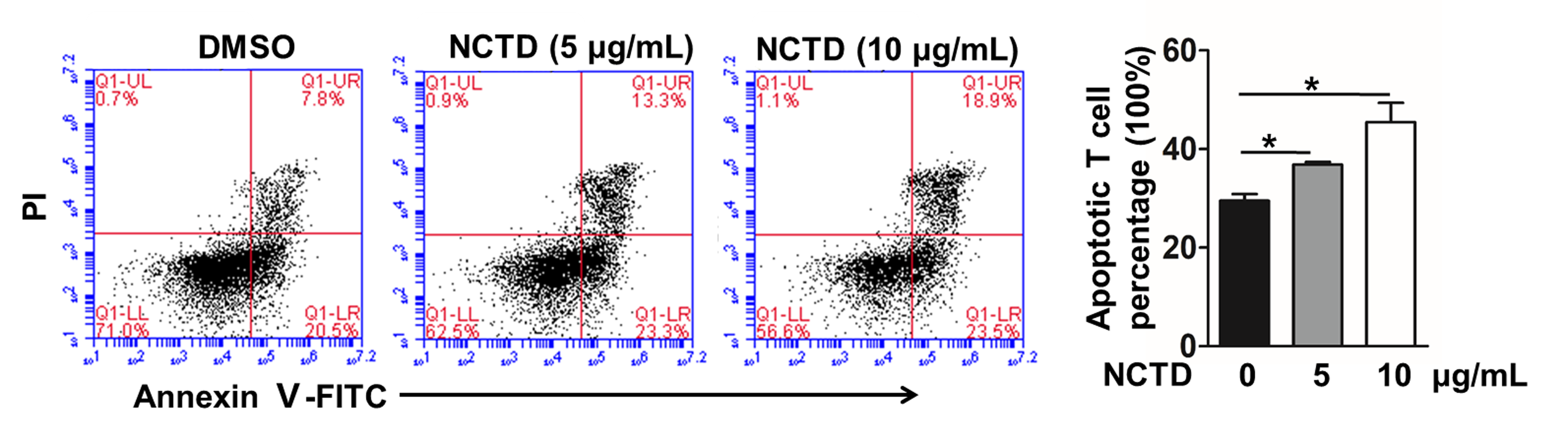

Supplement: Supplementary file 4 — SuppFig 4 [file 41401_2021_773_MOESM4_ESM.tiff]

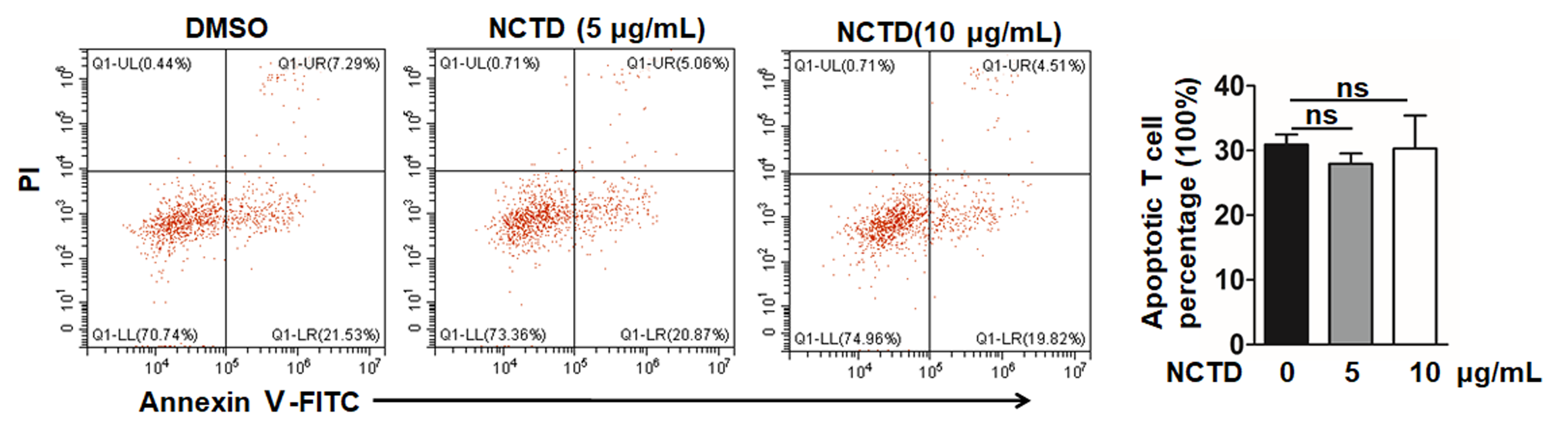

Supplement: Supplementary file 5 — SuppFig 5 [file 41401_2021_773_MOESM5_ESM.tif]
